# Modulation of energy metabolism to overcome drug resistance in chronic myeloid leukemia cells through induction of autophagy

**DOI:** 10.1038/s41420-022-00991-w

**Published:** 2022-04-20

**Authors:** Yiqing Li, Peiting Zeng, Jie Xiao, Peng Huang, Panpan Liu

**Affiliations:** 1 Guangdong Provincial Key Laboratory of Malignant Tumor Epigenetic and Gene Regulation, Sun Yat-Sen Memorial Hospital, Sun Yat-Sen University, Guangzhou, China; 2 Department of Hematology, Sun Yat-Sen Memorial Hospital, Sun Yat-Sen University, Guangzhou, China; 3 State Key Laboratory of Oncology in South China, Collaborative Innovation Center for Cancer Medicine, Sun Yat-sen University Cancer Center, 651 Dongfeng East Road, 510060 Guangzhou, China; 4 Department of Medical Oncology, Sun Yat-sen University Cancer Center, 651 Dong Feng East Road, 510060 Guangzhou, China

**Keywords:** Cancer metabolism, Chronic myeloid leukaemia

## Abstract

Tyrosine kinase inhibitors (TKIs) such as imatinib (IM) are key drugs for treatment of chronic myeloid leukemia (CML). Development of drug resistance to TKIs due to BCR-ABL mutation, especially T315I mutation, poses a major challenge in the clinical treatment of CML. The purpose of this study was to test metabolic modulation as a potential strategy to overcome imatinib resistance based on the possible crosstalk between BCR-ABL signaling and metabolic changes in CML. 2-deoxy-d-glucose (2-DG) was used to modulate the glucose metabolism in CML cells sensitive to IM (KBM5 cell line) and resistant to imatinib with BCR-ABL T315I mutation (KBM5-T315I cell line). Seahorse XFe24 extracellular flux analyzer to quantify oxygen consumption rate (OCR) and extracellular acidification rate (ECAR) was used to measure cellular energy metabolism. Cell proliferation was analyzed by CCK-8 assay and MTS assay. Annexin V/PI staining was used to evaluate cell apoptosis. Autophagy-related proteins and enzyme/proteins were detected by Western blotting. Cellular ATP concentration was detected using an ATP-based Cell Titer Kit. The combined action of 2-DG and IM was evaluated by calculating the drug combination index. Our results found that inhibition of glucose metabolism by 2-DG significantly impaired the viability of CML cells and co-treatment with 2-DG and imatinib induced a synergistic inhibition of KBM5 and KBM5-T315I cells. 2-DG induced cell death by autophagy, not by apoptosis, as evidenced by increased expression of Beclin1 and LC3AII and lack of annexin V/PI-positive cells. At the biochemical level, 2-DG inhibited glycolysis and mitochondrial oxygen consumption manifested by a significant decrease in ECAR and OCR, and a depletion of ATP. The severe metabolic stress induced by 2-DG in CML cells led to autophagic cell death. Our results suggested a metabolic vulnerability of CML cells that could be targeted by a combination of 2-DG and imatinib as an alternative treatment for imatinib-resistant CML.

## Introduction

Chronic myeloid leukemia (CML) is a myeloid proliferative disorder driven by constitutive activation of tyrosine kinase due to abnormal chromosome translocation with the formation of *BCR-ABL* (breakpoint cluster region-Abelson) fusion gene. Since abnormal activation of tyrosine kinase is the key molecular event that causes CML, tyrosine kinase inhibitors (TKIs) such as imatinib (IM) and its derivatives are mainstay of drugs for CML treatment and have significantly improved the clinical outcome of these leukemia patients during the past decades. However, many patients eventually develop TKI-resistance and relapse due in part to mutations at BCR-ABL. Among such mutations, T315I is a frequent mutation that causes significant resistance to TKIs [1]. Although the third-generation TKI ponatinib exhibits good therapeutic activity against T315I mutation, its clinical utility is somewhat limited due to its cardiovascular toxicity [2]. Thus, it is important to develop new therapeutic strategies that are effective against TKI-resistant CML cells based on their biological characteristics.

Alterations in energy metabolism are an important hallmark of cancer cells, and targeting the energy metabolism of cancer cells is considered as a promising strategy for cancer treatment [3, 4]. Our previous study showed that activation of tyrosine kinase due to BCR-ABL oncogenic signal could cause metabolic changes leading to a significant increase in ROS generation likely due to mitochondrial dysfunction [5]. A recent study showed that IM treatment of BCR-ABL-driven human leukemia cells led to modulation of glycolytic enzymes and reactivated mitochondrial oxidative phosphorylation [6]. Interestingly, the IM-resistant CML cells harboring BCR-ABL T315I mutation exhibited slow cell proliferation associated with down-regulation of glycolytic pathway and low ROS production [7]. These observations suggest that activation of tyrosine kinase could significantly impact cellular metabolism and affect drug sensitivity in leukemia cells. As such, modulation of cellular metabolism could be a potential strategy to enhance leukemia sensitivity to TKIs and overcome drug resistance.

The main goal of this study was to test the possibility to modulate glucose metabolism as a potential strategy to increase the sensitivity of TKI-resistant CML cells to IM treatment, using 2-deoxy-glucose (2-DG) as a tool to affect glucose metabolism. 2-DG is a known inhibitor of hexokinase (HK) and has been shown to enhance the sensitivity of tumor cells to certain chemotherapeutic drugs [8]. We found that 2-DG and IM had a synergistic inhibitory effect on the proliferation of CML cells, including cells harboring T315I mutation. Mechanistic study showed that 2-DG treatment caused a substantial decrease both in oxygen consumption rate (OCR) and extracellular acidification rate (ECAR) leading to a significant reduction in cellular ATP. These changes in energy metabolism in the leukemia cells resulted in major autophagic cell death in CML. Our study suggests that it is feasible to increase therapeutic activity of TKIs and overcome drug resistance in CML cells by modulating energy metabolism.

## Results

### Inhibition of glucose metabolism by 2-DG synergistically enhance IM therapeutic activity against CML cells

**Fig. 1:**
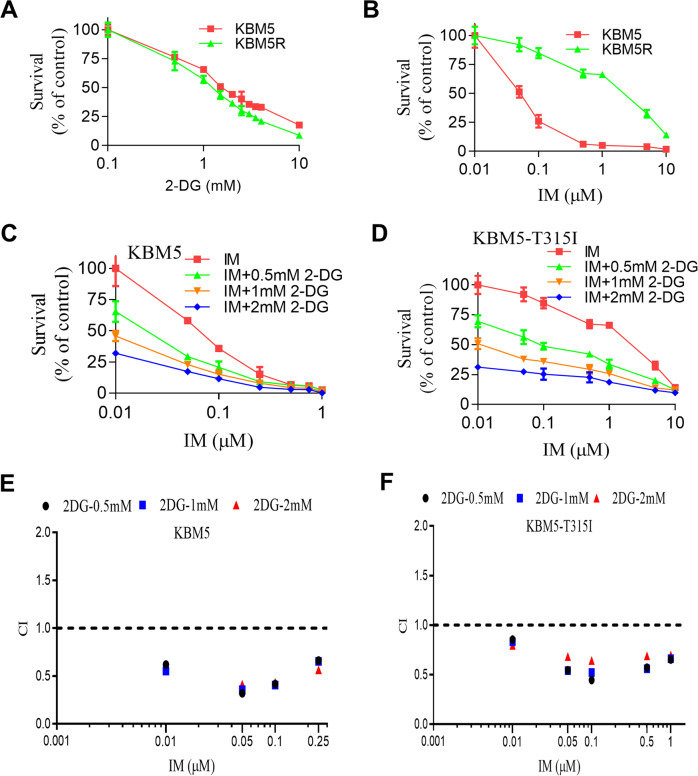
2-DG and Imatinib synergistically inhibit CML cell proliferation. **A**, **B** Dose-response curves of CML cells to 2-DG and IM. KBM5 and KBM5-T315I cells were treated with 0.1, 0.5, 1.0, 1.5, 2.0, 2.5, 3.0, 3.5, 4.0, 10 mM 2-DG or 0.01, 0.05, 0.1, 0.5, 1.0, 5.0, 10.0 μM IM for 72 h, then cell viability was measured by a CCK-8 assay. Data were mean ± SD (*n* = 3). **C**, **D** Dose-response curves of IM with or without 0.5, 1.0, 2.0 mM 2-DG for KBM5 and KBM5-T315I cells. Data were mean ± SD (*n* = 3). **E**, **F** Combination index (CI) of 2-DG and IM in the indicated two cell line cells described in (**C**, **D**) was analyzed by using CalcuSyn software. CI > 1 indicates antagonist effect; CI = 1 indicates additive effect; CI < 1 indicates synergistic effect.

Since our previous study suggested that BCR-ABL mutation, especially T315I, cause metabolic alterations associated with changes in drug sensitivity [5, 9], we first test the effect of 2-deoxyglucose (2-DG), a glycolytic inhibitor that target HK, on the cytotoxic effect of IM in CML cells with BCR-ABL (KBM5 cell line) and with T315I mutation (KBM5-T315I cells), using both CCK-8 assay and direct cell counting. As shown in Fig. 1A, B and Supplementary Fig. S1A, B, 2-DG and IM significantly inhibited the viability and the proliferation of KBM5 and KBM5-T315I cells in a dose-dependent manner. The IC_50_ values of IM were 0.09 μM and 2.84 μM for KBM5 cells and KBM5-T315I cells, respectively. The 30 folds difference in IC_50_ values for the two cell lines confirmed that T315I mutation conferred significant drug resistance to IM. In contrast, the IC_50_ values of 2-DG in KBM5 and KBM5-T315I cells were 1.62 mM and 1.28 mM, respectively. These similar IC_50_ values indicate that there was no co-resistance between IM and 2-DG likely due to their different mechanisms of action, and suggest a potential possibility to use 2-DG to overcome IM resistance. To test this possibility, we compared the drug combination (2-DG + IM) effect on KBM5 and KBM5-T315I cells, using cell viability as the assay end point. Cells were treated with various concentration of 2-DG (0, 0.5, 1, 2 mM) and IM (0, 0.05, 0.1, 0.25, 0.5, 0.75, 1.0 μM for KBM5 and 0, 0.05, 0.1, 0.5, 1.0, 5.0, 10.0 μM for KBM5-T315I) alone or in combination, and cell survival was subsequently measured by a CCK-8 assay to determine cytotoxicity. As shown in Fig. 1C, D, combination of 2-DG and IM resulted in a dose-dependent enhancement of inhibition of cell growth in both KBM5 and KBM5-T315I cells, indicating the ability of 2-DG to enhance the sensitivity of the drug-resistant KBM5-T315I cells to IM. Consistently, direct cell counting also showed that 2-DG enhanced the suppressive effect of IM on CML cell proliferation (Supplementary Fig S1C, D). Further quantitative analysis of drug combination indexes (CI values) between 2-DG and IM showed that the CI values were mostly <1.0 in both CML cell lines (Fig. 1E, F), indicating a strong synergy between the two drugs. To further test the high dependency of CML cells with T315I mutation on glycolysis, we compared KBM5 and KBM5-T315I cells for their sensitivity to IM with or without glucose starvation. As shown in Supplementary Fig S2A, glucose starvation alone could impair cell viability in both CML cell lines, with significantly more inhibition observed in KBM5-T315I cells. Consistently, we found that glucose starvation significantly sensitized the drug-resistant KBM5-T315I cells with mutation, whereas the parental KBM5 cells remained sensitive toIM with or without glucose (Supplementary Fig S2B, C).

### 2-DG exerts its anticancer effect against CML cells through induction of autophagy

**Fig. 2:**
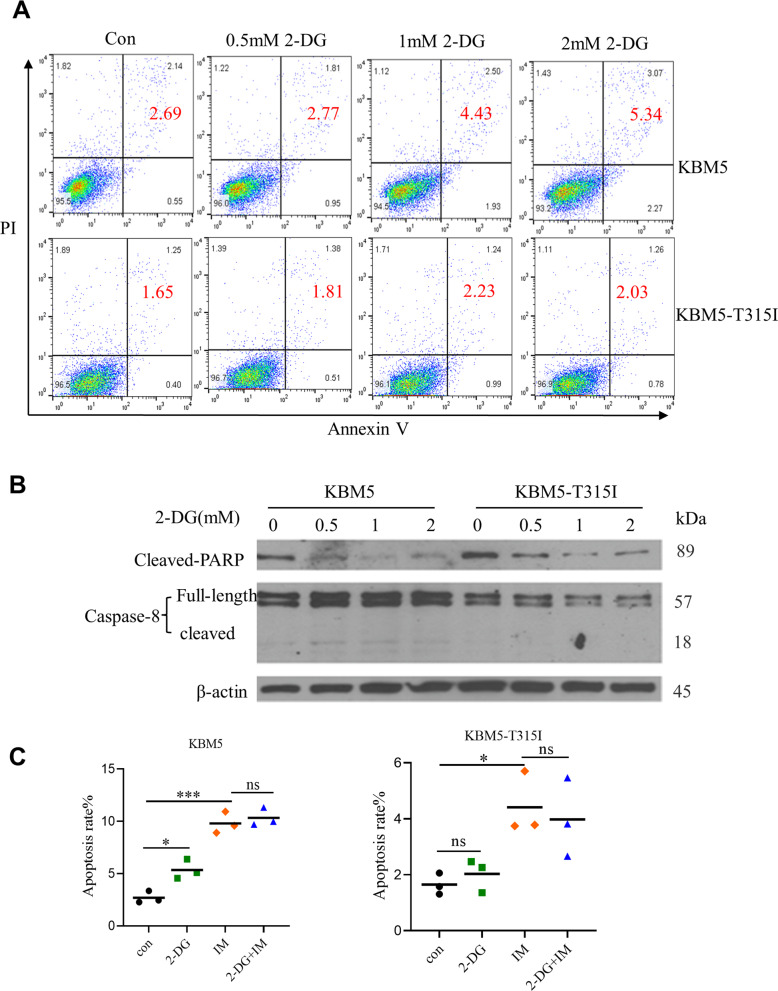
2-DG induces CML cell death in an apoptosis-independent pathway. **A** KBM5 and KBM5-T315I cells were treated with indicated concentrations of 2-DG for 48 h, and cell viability was measured by Annexin V-FITC/PI staining and flow cytometry analysis. The result from a representative experiment is shown. The number (%) within each flow cytometry panel indicates % of dead cells (Annexin V-FITC/PI double-positive). **B** Western blotting analysis of cleaved PARP and cleaved/full-length caspase-8 before and after 2-DG treatment. KBM5 and KBM5-T315I cells were treated with 2-DG at the indicated concentrations for 48 h, and proteins were isolated for western blotting analysis of cleaved-PARP, cleaved/full-length caspase-8 and β-actin. **C** Apoptosis analysis of CML cells in response to 2-DG and/or IM treatment. Quantitative results of three separate experiments are shown. Each bar shows mean ± SD, *P < 0.05, ***P < 0.001, ns not significant.

Apoptosis is a common mode of cell death in leukemia cells treated with anticancer drugs. To test if 2-DG could induce apoptosis in CML cells, we performed flow cytometry analysis using Annexin V-FITC/PI. We found 2-DG, although effective in abrogating cell survival (Fig. 1A), did not induce detectable apoptosis at the concentrations up to 2 mM (Fig. 2A). Consistently, western blotting analysis of apoptotic markers such as cleavage of caspase-8 and PARP (poly ADP-ribose polymerase) showed that these apoptotic molecular events did not occur when KBM5 and KBM5-T315I cells were treated with 2-DG (Fig. 2B). These results together suggest 2-DG induced cell death was not mediated by the apoptotic pathway. We then examined if 2-DG in combination with IM could induced apoptosis in KBM5 or KBM5-T315I cells. KBM5 cells were treated by 2 mM 2-DG combined with 0.1 μM IM and KBM5-T351I cells were treated by 2 mM 2-DG combined with 5.0 μM IM. As shown in Fig. 2C, there was only a slight increase in apoptosis (~5–10%) induced by IM alone, addition of 2-DG could not further enhance IM-induced apoptosis.

**Fig. 3:**
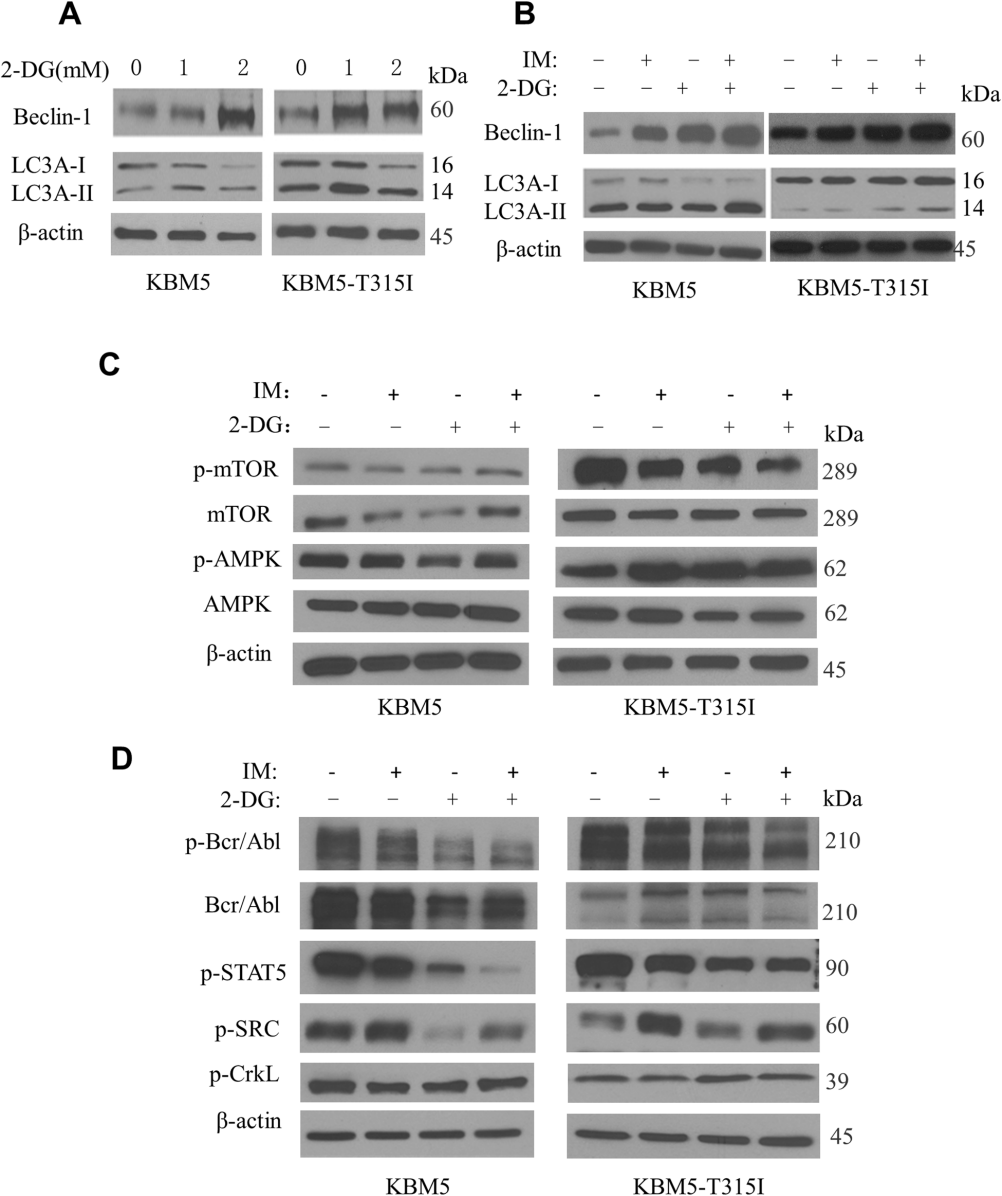
2-DG in combination with imatinib induces autophagy in CML cells. **A** KBM5 and KBM5-T315I cells were treated with 2-DG at indicated concentrations for 48 h, and proteins were isolated for western blot analysis of beclin-1, LC3A-I/II and β-actin. **B** KBM5 and KBM5-T315I cells were treated with 2-DG (2 mM) and/or IM (0.1 μM for KBM5 cells and 5 μM for KBM5-T315I cells) for 48 h, and proteins were isolated for western blot analysis of beclin-1, LC3A-I/II and β-actin. **C** KBM5 and KBM5-T315I cells were treated with 2-DG (2 mM) and/or IM (0.1 μM for KBM5 cells and 5 μM for KBM5-T315I cells) for 48 h, and proteins were isolated for western blot analysis of p-mTOR, mTOR, p-AMPK, AMPK and β-actin. **D** KBM5 and KBM5-T315I cells were treated with 2-DG (2 mM) and/or IM (0.1 μM for KBM5 cells and 5 μM for KBM5-T315I cells) for 48 h, and proteins were isolated for western blot analysis of p-Bcr/Abl, Bcr/Abl, p-STAT5, p-SRC, p-CrkL, and β-actin.

As the cytotoxic effect of 2-DG seemed not due to the induction of apoptosis, we then investigated if 2-DG could induce autophagy via its inhibitory effect on energy metabolism. Western blotting was used to analyze two autophagy-related proteins, beclin-1 and LC3A (microtubule-associated protein 1 light chain 3A). As shown in Fig. 3A, treatment with 2-DG enhanced the expression levels of beclin-1, and promoted to conversion of LC3AI to LC3A-II in both KBM5 and KBM5-T315I cell lines. Combination of 2-DG and IM also increased the expression levels of beclin-1 and LC3A-II (Fig. 3B). Since the AMPK and mTOR signaling pathways play a role in autophagy regulation, we tested if 2-DG and IM-induced autophagy might be associated with any changes in AMPK and mTOR. As shown in Fig. 3C, there was a decrease in mTOR phosphorylation associated with an increased AMPK phosphorylation in KBM5-T315I cells after treatment with IM and 2-DG. Interestingly, such changes were not observed in the parental KBM5 cells. Consistently, we also observed a decrease in mTOR phosphorylation and an increase in AMPK phosphorylation in cells under glucose starvation and treated with IM. Moreover, phosphorylation of p70 S6 kinase (p-p70S6K), a downstream molecule of the AMPK-mTOR pathway, decreased significantly (Supplementary Fig S3). Since the mTOR–70S6K pathway was recently shown to be involved in autophagy [10], our data together suggest that abrogation of glycolysis could induce autophagy via the AMPK-mTOR-70S6K signaling.

We also examined the expression changes in BCR/ABL protein and molecules associated with the BCR/ABL signaling, including p-CrkL, p-STAT5, and p-SRC. In KBM5 cells, the levels of Bcr-Abl phosphorylation and protein content were substantially reduced, indicating an inhibition of Bcr-Abl phosphorylation and a decrease in protein stability (Fig. 3D, left panel). Consistently, the phosphorylation of the downstream molecules p-CrkL, p-STAT5, and p-SRC were also decreased by treatment with 2-DG and IM in KBM5 cells (Fig. 3D, left panel). In the IM-resistant KBM5-T315I cells, no significant changes in Bcr-Abl and its downstream proteins were observed (Fig. 3D, right panel).

### Co-treatment with 2-DG and IM induce a severe energy crisis in CML cells

**Fig. 4:**
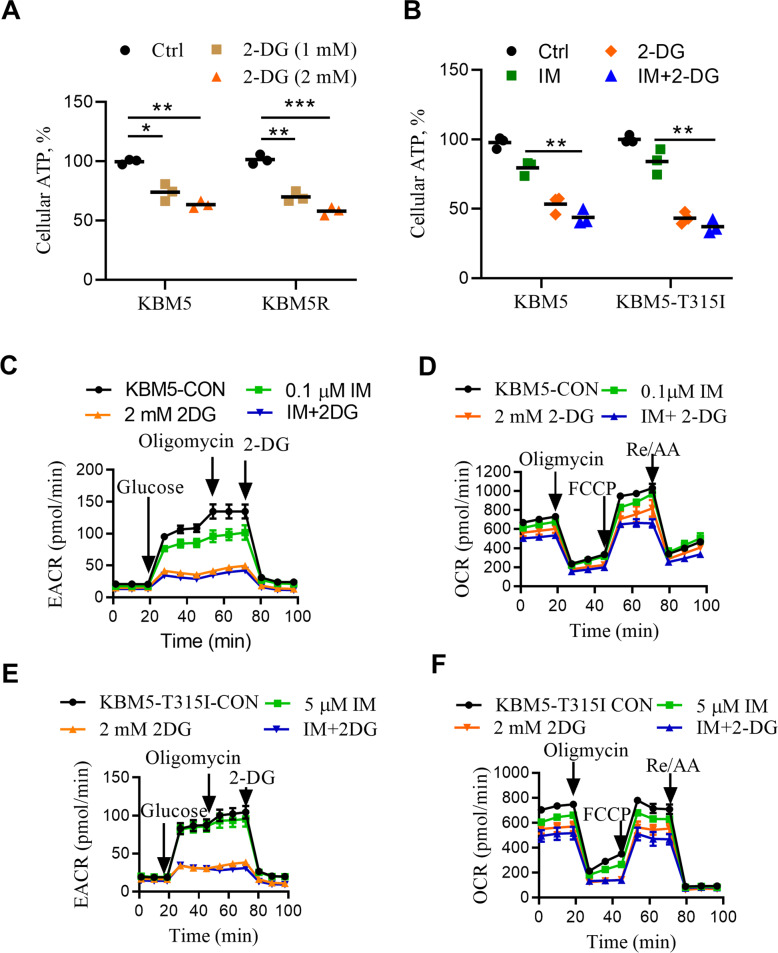
Effect of 2-DG and imatinib on cellular energy metabolism. **A** KBM5 and KBM5-T315I cells were treated with 2-DG at indicated concentrations for 24 h, and intracellular ATP levels were measured. Each bar represents means ± SD, *n* = 3, *P < 0.05, **P < 0.01, ***P < 0.001. **B** KBM5 and KBM5-T315I cells were treated with 2-DG (2 mM), IM (0.1 μM for KBM5 cells and 5 μM for KBM5-T315I cells) and their combination for 24 h, and intracellular ATP levels were measured. Each bar represents means ± SD, *n* = 3, **P < 0.01. **C–F** Real-time measurement of OCR and ECAR in indicated conditions using the Seahorse Bioscience Extra Cellular Flux Analyzer. For the glycolysis analysis (**C**, **E**), cells were firstly treated with 2-DG (2 mM), IM (0.1 μM for KBM5 cells and 5 μM for KBM5-T315I cells) and their combination for 6 h, and then cells were sequentially treated with glucose, oligomycin (O, 1 μM), 2-DG (1 μM).For the mitochondrial function analysis (**D**, **F**), cells were firstly treated with 2-DG (2 mM), IM (0.1 μM for KBM5 cells and 5 μM for KBM5-T315I cells) and their combination for 6 h, and then sequentially treated with, oligomycin (O, 1 μM), FCCP (F, 1 μM) and rotenone/antimycin A (R/A, 0.5 μM).

Autophagy is a cellular process that respond to metabolic stress [11], which could protect the tumor cells under temporal nutrient deprivation or could lead to cell death if the stress persists. As 2-DG could inhibit glucose phosphorylation by HK, we hypothesized that this inhibition could suppress cellular ATP generation from glucose, leading to activation AMPK and induction of autophagy. To test this hypothesis, we measured ATP production in KBM5 and KBM5-T315I cells treated with 2-DG, and found a major depletion of ATP after 2-DG treatment in both cell lines (Fig. 4A). We then tested the combination effect of 2-DG and IM on ATP production. As shown in Fig. 4B, treatment of CML cells with IM alone did not cause a significant ATP reduction, whereas combination of 2-DG and IM resulted in a severe ATP depletion. Since ATP is mainly produced by oxidative phosphorylation in the mitochondria and glycolysis in the cytosol, we then used Seahorse XFe24 extracellular flux analyzer to measure oxygen consumption rate (OCR, a key indicator of oxidative phosphorylation) and extracellular acidification rate (ECAR, an indicator of glycolysis). In both CML cell lines, 2-DG caused a major inhibition in glycolysis, as indicated by a significant decrease in ECAR in the presence of glucose (Fig. 4C, E, Supplementary Fig S4), consistent with the ability of 2-DG to inhibit the glycolytic enzyme HK. Importantly, such glycolytic inhibition did not induce a compensatory increase of mitochondrial oxygen consumption, as indicated by the lack of OCR increase and no compensatory increase in mitochondrial ATP production when the cells were treated with 2-DG (Fig. 4D, F, Supplementary Fig S4). Instead, OCR was slightly reduced by 2-DG in both CML cell lines, likely reflecting the decrease of pyruvate as the fuel for mitochondrial oxidative phosphorylation due to reduced glycolysis as the consequence of inhibition of HK by 2-DG. The suppression of glycolysis and the subsequent decrease in mitochondrial oxidative phosphorylation might explain why 2-DG was effective in causing ATP depletion in CML cells leading to autophagic cell death in CML.

### Co-treatment of CML cells with 2-DG and IM down-regulates the glucose transporters and hexokinase II but not mitochondrial ETC components

**Fig. 5:**
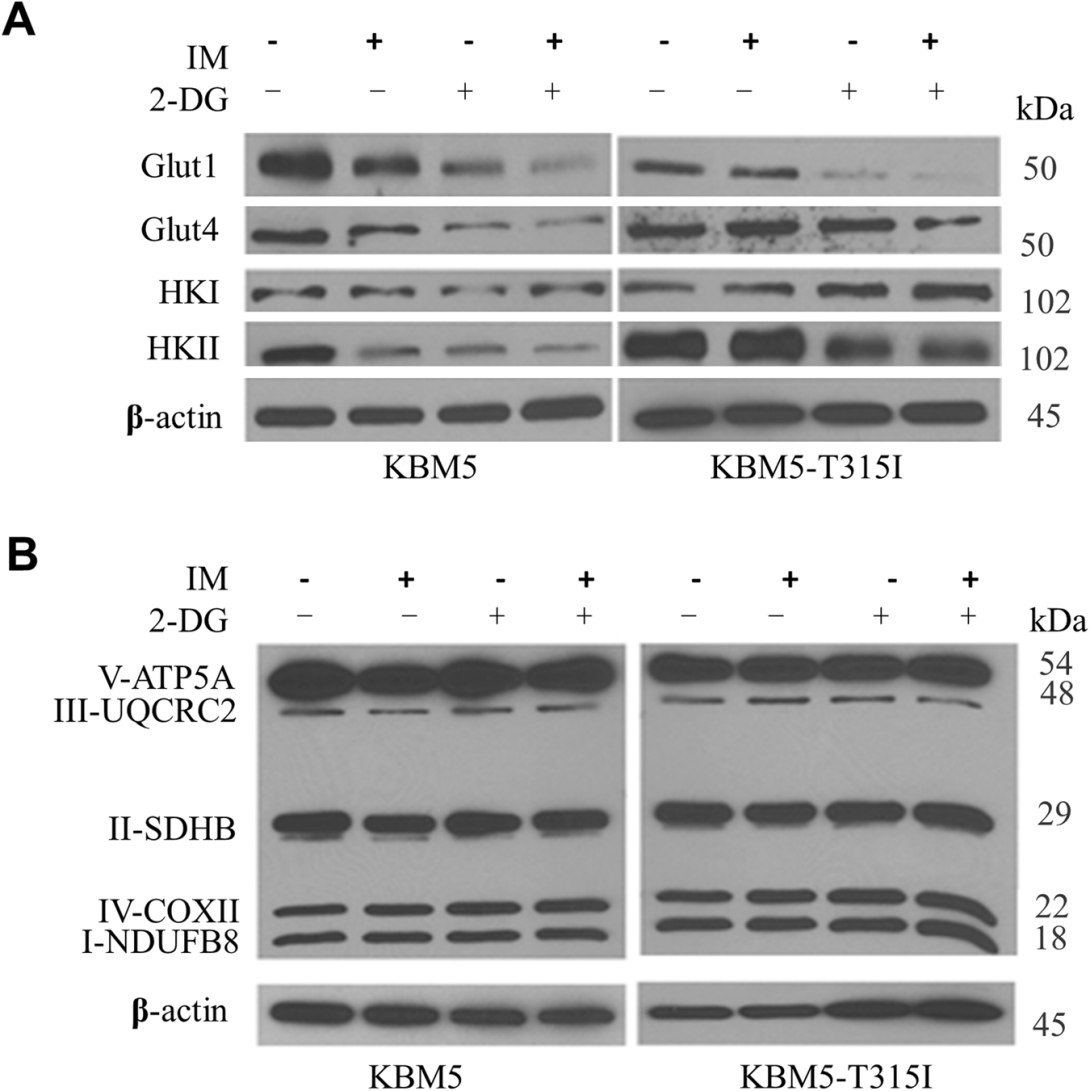
Effect of 2-DG and imatinib on the expression of glucose transporters, hexokinases and mitochondrial ETC components. **A** KBM5 and KBM5-T315I cells were treated with 2-DG (2 mM) and/or IM (0.1 μM for KBM5 cells and 5 μM for KBM5-T315I cells) for 48 h, and proteins were isolated for western blot analysis of Glut-1, Glut4, HKI, HKII and β-actin. **B** KBM5 and KBM5-T315I cells were treated with 2-DG (2 mM) and/or IM (0.1 μM for KBM5 cells and 5 μM for KBM5-T315I cells) for 48 h, and proteins were isolated for western blot analysis of five key complexes of OXPHOS and β-actin.

Cellular glucose metabolism begins with the import of glucose into cells primarily via glucose transporters (Gluts) followed by phosphorylation to glucose-6-phosphate (G-6-P) by HKs [12]. We analyzed the expression levels of Glut1, Glut4, HKI and HKII in CML cells treated with 2-DG and IM alone or in combination. The results showed that 2-DG and its combination with IM caused a down-regulation of glucose transporters Glut1 and Glut4, and a major decrease in glycolytic enzyme HKII in both KBM5 and KBM5-T315I cells (Fig. 5A). Such decrease was most significant when CML cells were treated with both compounds. Next, we tested the effect of 2-DG and IM on the expression levels of the mitochondrial electron transport chain (ETC) components, using Western blotting analysis with cocktail antibodies against ETC. The results revealed no detectable changes of ETC expression in KBM5 or KBM5-T315I cells following exposed to 2-DG and IM for 48 h (Fig. 5B), suggesting that the inhibitory effect of IM and 2-DG on OCR in CML cells was mainly due to the lack of metabolic substrate (pyruvate) rather than changes in mitochondrial ETC expression.

## Discussion

Resistance to TKIs is a key feature of poor prognosis in CML, and poses a major challenge in clinical treatment of this disease. Development of new strategies to effectively overcome resistance to TKIs remains as an urgent need in CML management [13]. Targeting the metabolic changes in CML with BCR-ABL mutation is a potentially effective strategy. Previous study has revealed significant metabolic changes such as high ROS generation and more dependence on active glycolysis in CML cells with BCR-ABL mutation [5, 7, 9, 14]. These observations provide a biochemical basis for metabolic intervention to impact drug-resistant CML cells with drugs that inhibit glucose metabolism. 2-DG is a glucose analog that is taken up by cells through glucose transporters and phosphorylated by HK [15]. The phosphorylated product 2-D-glucose-6-phosphate cannot be further metabolized, and acts as an inhibitor of HK. This compound has been considered as an anticancer agent, and in vivo studies suggest that it was well tolerated when administered in combination with other therapy [16, 17]. The anticancer effect of 2-DG in acute lymphoblastic leukemia cells has also been reported [18, 19]. However, its potential therapeutic activity against CML cells still remains unclear.

In the present study, we found that 2-DG was effective in inhibition of CML cells harboring BCR-ABL with or without T315I mutation. This is consistent with the observation that CML cells with *BCR-ABL* fusion gene tend to shift their energy metabolism toward active glycolysis [6, 20]. An inhibition of this key energy metabolic pathway would compromise the viability of CML cells and impair their ability to sustain the cytotoxic impact of IM. Our study using two experimental approaches, either with pharmacological inhibitor 2-DG or with glucose-starvation method, demonstrated that CML cells with T315I mutation were more dependent on glucose metabolism and thus more sensitive to glycolytic inhibition or glucose depletion. Interestingly, 2-DG seems to exert its cytotoxic effect against CML cells mainly by induction of autophagy without significant apoptosis, as evidenced by an induction of LC3A-II expression, the lack of annexin-V/PI staining, and no cleavage of caspase-8 and PARP. This is in contrast to the cell death induced by many chemotherapeutic agents, which often cause apoptosis in leukemia [21–23]. The autophagic cell death induced by 2-DG was likely due to metabolic stress as the consequence of glycolytic inhibition leading to ATP depletion. Interestingly, recent studies suggest that 2-DG could inhibit N-glycosylation and induce endoplasmic reticulum stress, and might contribute to its antiproliferative effect against breast cancer cells and acute myeloid leukemia cells [24, 25]. Thus, it is possible that the impact of 2-DG on CML cells observed in our study could also be mediated by multiple mechanisms.

During nutrient deficiency, autophagy functions as a pro-survival mechanism. However, sustained and elevated autophagy may lead to cell death and suppress tumorigenesis [26]. Indeed, previous studies showed that 2-DG could induce autophagy by mimicking effect of glucose deprivation [27–29]. However, it is unclear if such autophagic response could help the cancer cells to sustain the insult by other anticancer agents (pro-survival) or could enhance drug-induced cell death. It is extremely important to distinguish these two different possibilities, which would lead to opposite decision regarding the combination of 2-DG with other anticancer drugs. In the case of combination with IM, our study clearly showed that 2-DG promoted autophagy in CML cells and significantly enhanced the sensitivity of the leukemia cells to IM. Induction of autophagy by 2-DG was evidenced by the conversion of the cytoplasmic form of LC3 (LC3-I) to the phosphatidylethanolamine-LC3 conjugate (LC3- II) and the increased expression of Beclin1, two typical markers of autophagy [30, 31]. The significant enhancement of IM-induced cytotoxicity by 2-DG is supported quantitatively by the calculated CI values, which were <1.0 indicating synergy between the two drugs. Cellular metabolism is closely associated with autophagy [32]. Previous studies showed that 2-DG could competitively inhibit glucose uptake and impact both glycolysis and OXPHOS [33]. The results of our study using XFe24 extracellular flux analyzer to measure OCR and ECAR support the conclusion that 2-DG could inhibit glycolysis and subsequently reduces mitochondrial oxidative phosphorylation likely due to a decrease in supply of pyruvate. This could explain why 2-DG was effective in causing severe ATP depletion leading to autophagic death of CML cells. Of particular note, a change in ATP might significantly affect the AMPK pathway, which is known to promote autophagy through direct phosphorylation of the mammalian autophagy-initiating kinase (Ulk1) [34]. As an energy sensor, AMPK activity is closely regulated by the ratio of AMP/ATP in the cells, with an increase of this ratio being a strong activation signal for AMPK [35]. In CML cells, the significant decrease of ATP induced by 2-DG would likely cause an increase of AMP/ATP ratio, leading to AMPK activation and subsequently the occurrence of autophagy. In this context, it would be of interest to measure cellular AMP level in 2-DG treated CML cells to determine the actual change of AMP/ATP ratio.

The ability of 2-DG to inhibit glycolysis could be explained by its direct competition with glucose for transport into the cells and its direct inhibition of HKs. The mechanism by which 2-DG reduces mitochondrial oxidative phosphorylation seems mainly due to metabolic substrate limitation (e.g., a decrease in pyruvate due to glycolytic inhibition), and not due to a direct inhibition of mitochondrial function per se, since there were not changes in expression of the mitochondrial ETC components after 2-DG treatment. Inhibition of glycolysis by 2-DG could cause a decrease of pyruvate, and thus limit its availability to fuel mitochondrial oxidative phosphorylation. To further elucidate the detail changes in specific metabolic pathways induced by 2-DG and IM in CML cells, it would be of significant interest to use [U-^13^C]-glucose and [U-^13^C]-glutamine in metabolic flux analyses to quantitatively dissect the detail changes affected by 2-DG and IM in future study.

CML patients with T315I mutation often have poor clinical outcome due to resistance to standard treatment with IM and possibly other second generation of TKIs. A recent study has discovered a potent pan-inhibitor of BCR-ABL kinase capable of inhibiting the T315I-resistant mutant [36], its feasibility to enter clinical development for potential use in CML treatment still requires further study. The ability of 2-DG to effectively inhibit CML cells with T315I mutation and enhance their sensitivity to IM is of particular importance. Since T315I mutation is a major molecular mechanism leading to IM resistance in CML patients, it would be possible to use 2-DG in combination with IM to overcome drug resistance due to T315I mutation. A previous clinical study showed that 2-DG is safe and could be tolerated in glioblastoma patients without any acute toxicity [37]. Considering the safety profiles of both 2-DG and IM in cancer patients, it seems feasible to test the combination of 2-DG and IM for treatment of drug-resistant CML in clinical trials. Since currently there are limited treatment options for CML patients who have developed drug resistance to TKIs, testing the therapeutic efficacy of 2-DG and IM combination in CML patients with T315I mutation merits further consideration.

In summary, we showed that inhibition of glycolysis and oxidative phosphorylation by 2-DG could significantly impair the viability of CML cells, and enhance their sensitivity to IM. 2-DG induced CML cell death by autophagy, not by apoptosis. Mechanistically, 2-DG inhibited glycolysis and mitochondrial energy metabolism lead to a severe depletion of ATP in CML cells leading to autophagic cell death. Combination of 2-DG and IM caused a synergistic inhibition against CML cells, including those with T315I mutation that were otherwise resistant to IM alone. Our study has identified a metabolic vulnerability of CML cells that could be targeted by a combination of 2-DG and IM to increase therapeutic activity and overcome resistance.

## Materials and methods

### Cell culture and reagents

The human CML cell line KBM5 (harboring BCR-ABL) and its IM-resistant sub-line with T315I mutation (KBM5-T315I cells) were cultured at 37 °C with 5% CO_2_ in Iscove’s Modified Dulbecco’s Media (IMDM, from Gibco, USA) supplemented with 10% fetal bovine serum (FBS, from Hyclone, USA) as we described previously [9]. KBM5-T315I cells were routinely maintained in IMDM medium with IM (1.0 μM), which was removed 2–3 days before KBM5-T315I cells were used for experiments. IM was purchased from Selleckchem (Houston, TX, USA) and 2-DG was obtained from Cayman Chemical (Ann Arbor, MI, USA). Annexin V-FITC/PI apoptosis kit was from BD Biosciences (San Jose, CA, USA). Seahorse base medium, oligomycin, Carbonyl cyanide-p-trifluoromethoxy phenylhydrazone (FCCP), and rotenone/antimycin A were obtained from Seahorse Bioscience (North Billerica, MA, USA). Antibodies against AMPK(#5832), p-AMPK(#2537), Bcr/Abl(#3908), p-Bcr/Abl (#3901),beclin-1 (#4122), intact and cleaved caspase-8 (#9748, #9746), cleaved PARP (#5625), Glut1 (#12939), Glut4 (#2213), HK I (#2024), HK II (#2106), LC3A (#4599), mTOR (#4517), p-mTOR (#5536), p-p70S6K (Thr389) (#97596), p70S6K (#34475), p-CrkL (#3181), p-SRC (#2101), p-STAT5 (#4322), β-actin (#3700) were purchased from Cell Signaling Technology (Beverly, MA, USA). Antibody to total OXPHOS cocktail (#110411) was purchased from Abcam (Cambridge, MA, USA).

### Cell viability and proliferation assays

Cell viability was measured using cell counting kit-8 (CCK-8 from Dojindo, Kumamoto, Japan) or MTS (3-(4,5-dimethylthiazol-2-yl)-5-(3-carboxymethoxyphenyl)-2-(4-sulfophenyl)-2H-tetrazolium, inner salt and PMS (phenazine methosulfate)) (Promega, Madison, WI, USA) assay according to the manufacturer’s instructions. 2 × 10^4^ cells were seeded in 96-well plates and treated with 2-DG and/or IM for 72 h. Then CCK-8 or MTS was added and incubated for 4 h at 37 °C. The spectrometric absorbance was measured at 470 nm for CCK-8 or 490 nm for MTS by a microplate reader (Multiskan GO,Thermo Scientific, USA).

To measure cell proliferation, 1.5 × 10^5^ cells were seeded into 12-well plates and treated with the indicated concentrations of 2-DG and/or IM for 72 h. Cell numbers were directly counted at the end point, using an automated cell counter (Cellometer AutoT4, Nexcelom, USA).

### Apoptosis analysis

Cells were treated with 2-DG and/or IM, collected and stained with Annexin V-FITC/PI according to the instructions of the manufacturer. Stained cells were evaluated by flow cytometry (FACS Calibur- Becton Dickinson, Franklin Lakes, NJ, USA) and data were analyzed using FlowJo software package (Tree Star, Inc., Ashland, OR, USA). Each experiment was repeated at least three times.

### Measurement of cellular ATP

Cellular ATP concentration was detected using an ATP-based Cell Titer-Glo Luminescent Cell Viability Kit according to the manufacturer’s instructions. A total of 2 × 10^5^ cells were plated in 96-well plates and treated with drugs for 24 h. Then, 100 μL Cell Titer-Glo reagents were added to each well and rocked for 2 min to lyse cell. The samples were kept at room temperature for 10 min. The ATP contents were recorded as luminescent signal, using a luminescent plate reader (Thermo Fisher, VarioskanFlash, Waltham, MA, USA).

### Western blotting analysis

Cells were lysed with lysing buffer. The proteins were quantified, equally loaded on the gel, and separated in 8% or 12% polyacrylamide gels. The proteins were then transferred to the PVDF membranes, which were incubated with primary antibodies for overnight at 4 °C, followed by incubation with horseradish peroxidase-conjugated secondary antibodies for 1 h at room temperature. Finally, the immunoreactive bands were detected by ECL buffer and exposed to X-ray film (Kodak, Tokyo, Japan). The original western blot images are shown in Supplemental material-original western blots.

### Real-time cell metabolism assay

XFe24 Extracellular Flux Analyzer (Seahorse Bioscience, North Billerica, MA, USA) was used for real-time analysis of OCR and ECAR according to the manufacturer’s user guide. Cells were seeded overnight in a Seahorse 24-well culture microplate precoated by Cell-Tak. Then the medium was changed to pre-warmed Seahorse base medium supplemented with 1 mM pyruvate, 2 mM glutamine, and 10 mM glucose on the day of the assay and incubated in CO_2_-free incubator at 37 °C for 1 h prior to the assay. Injections of oligomycin (1 μM), FCCP (1 μM), and rotenone/antimycin A (0.5 μM) were loaded onto ports A, B and C respectively for OCR. Injections of glucose (10 mM), oligomycin (2 μM), and 2-DG (100 mM) were loaded onto ports A, B and C respectively for ECAR. Results were normalized to cell number. All experiments were performed three times independently.

### Statistical analysis

Statistical analysis was performed using SPSS 17.0 software and GraphPad Prism software (GraphPad Software, Inc, San Diego, CA, USA). All the data were presented as the means ± SD. One-way ANOVA was used to assess the significance of difference among multiple groups. *P* < 0.05 was considered as statistically significant. CompuSyn (Paramus, NJ, USA) was used to test the interaction of the drug combinations for synergy/additivity/antagonism using the CI value from the Chou-Talalay method. CI values of <1, 1, and >1 indicate synergistic, additive, and antagonistic effects, respectively [38].

## Supplementary information

Supplemental Figure S1

Supplemental Figure S2

Supplemental Figure S3

Supplemental Figure S4

Supplemental material-original western blots

## Data Availability

All data included in this study are available upon request by contact with the corresponding author.

## References

[1] Hochhaus A, Larson RA, Guilhot F, Radich JP, Branford S, Hughes TP (2017). Long-Term Outcomes of Imatinib Treatment for Chronic Myeloid Leukemia. N Engl J Med.

[2] Chan O, Talati C, Isenalumhe L, Shams S, Nodzon L, Fradley M (2020). Side-effects profile and outcomes of ponatinib in the treatment of chronic myeloid leukemia. Blood Adv..

[3] Zhu J, Thompson CB (2019). Metabolic regulation of cell growth and proliferation. Nat Rev Mol Cell Biol..

[4] Pavlova NN, Thompson CB (2016). The Emerging Hallmarks of Cancer Metabolism. Cell Metab..

[5] Trachootham D, Zhou Y, Zhang H, Demizu Y, Chen Z, Pelicano H (2006). Selective killing of oncogenically transformed cells through a ROS-mediated mechanism by beta-phenylethyl isothiocyanate. Cancer Cell.

[6] De Rosa V, Monti M, Terlizzi C, Fonti R, Del Vecchio S, Iommelli F. Coordinate Modulation of Glycolytic Enzymes and OXPHOS by Imatinib in BCR-ABL Driven Chronic Myelogenous Leukemia Cells. Int J Mol Sci. 2019;20:3134. 10.3390/ijms20133134 PMC6651622 31252559

[7] Ko BW, Han J, Heo JY, Jang Y, Kim SJ, Kim J (2016). Metabolic characterization of imatinib-resistant BCR-ABL T315I chronic myeloid leukemia cells indicates down-regulation of glycolytic pathway and low ROS production. Leuk. Lymphoma.

[8] Pelicano H, Martin DS, Xu RH, Huang P (2006). Glycolysis inhibition for anticancer treatment. Oncogene.

[9] Zhang H, Trachootham D, Lu W, Carew J, Giles FJ, Keating MJ (2008). Effective killing of Gleevec-resistant CML cells with T315I mutation by a natural compound PEITC through redox-mediated mechanism. Leukemia.

[10] Dai H, Hu W, Zhang L, Jiang F, Mao X, Yang G (2021). FGF21 facilitates autophagy in prostate cancer cells by inhibiting the PI3K-Akt-mTOR signaling pathway. Cell Death Dis.

[11] Li D, Song JZ, Shan MH, Li SP, Liu W, Li H (2015). A fluorescent tool set for yeast Atg proteins. Autophagy.

[12] Holman GD (2018). Chemical biology probes of mammalian GLUT structure and function. Biochem J..

[13] Braun TP, Eide CA, Druker BJ (2020). Response and Resistance to BCR-ABL1-Targeted Therapies. Cancer Cell.

[14] Ju HQ, Zhan G, Huang A, Sun Y, Wen S, Yang J (2017). ITD mutation in FLT3 tyrosine kinase promotes Warburg effect and renders therapeutic sensitivity to glycolytic inhibition. Leukemia.

[15] Leung K. 99mTc-Ethylenedicysteine-deoxyglucose. In: Molecular Imaging and Contrast Agent Database (MICAD) [Internet]. Bethesda (MD): National Center for Biotechnology Information (US); 2004–2013. 2009. [updated 2010Feb 16].

[16] Saleem A, Dvorzhinski D, Santanam U, Mathew R, Bray K, Stein M (2012). Effect of dual inhibition of apoptosis and autophagy in prostate cancer. Prostate.

[17] Prasanna VK, Venkataramana NK, Dwarakanath BS, Santhosh V (2009). Differential responses of tumors and normal brain to the combined treatment of 2-DG and radiation in glioablastoma. J Cancer Res Ther..

[18] Boag JM, Beesley AH, Firth MJ, Freitas JR, Ford J, Hoffmann K (2006). Altered glucose metabolism in childhood pre-B acute lymphoblastic leukaemia. Leukemia.

[19] Hulleman E, Kazemier KM, Holleman A, VanderWeele DJ, Rudin CM, Broekhuis MJ (2009). Inhibition of glycolysis modulates prednisolone resistance in acute lymphoblastic leukemia cells. Blood.

[20] Wierenga ATJ, Cunningham A, Erdem A, Lopera NV, Brouwers-Vos AZ, Pruis M (2019). HIF1/2-exerted control over glycolytic gene expression is not functionally relevant for glycolysis in human leukemic stem/progenitor cells. Cancer Metab..

[21] DeFeudis P, D’lncalci M, Broggini M (1996). Block of bcr-abl expression and induction of apoptosis by cisplatinum in a human chronic myeloid leukaemia cell line. Apoptosis.

[22] Stoetzer OJ, Pogrebniak A, Scholz M, Pelka-Fleischer R, Gullis E, Darsow M (1999). Drug-induced apoptosis in chronic lymphocytic leukemia. Leukemia.

[23] Saxena K, Konopleva M (2020). An expert overview of emerging therapies for acute myeloid leukemia: novel small molecules targeting apoptosis, p53, transcriptional regulation and metabolism. Expert Opin Investig Drugs.

[24] Berthe A, Zaffino M, Muller C, Foulquier F, Houdou M, Schulz C (2018). Protein N-glycosylation alteration and glycolysis inhibition both contribute to the antiproliferative action of 2-deoxyglucose in breast cancer cells. Breast Cancer Res Treat..

[25] Hu X, Chen F (2019). Targeting on glycosylation of mutant FLT3 in acute myeloid leukemia. Hematology.

[26] Prieto P, Rosales-Mendoza CE, Terrón V, Toledano V, Cuadrado A, López-Collazo E (2015). Activation of autophagy in macrophages by pro-resolving lipid mediators. Autophagy.

[27] Giammarioli AM, Gambardella L, Barbati C, Pietraforte D, Tinari A, Alberton M (2012). Differential effects of the glycolysis inhibitor 2-deoxy-D-glucose on the activity of pro-apoptotic agents in metastatic melanoma cells, and induction of a cytoprotective autophagic response. Int J Cancer.

[28] Ben Sahra I, Tanti JF, Bost F (2010). The combination of metformin and 2-deoxyglucose inhibits autophagy and induces AMPK-dependent apoptosis in prostate cancer cells. Autophagy.

[29] Xi H, Kurtoglu M, Liu H, Wangpaichitr M, You M, Liu X (2011). 2-Deoxy-D-glucose activates autophagy via endoplasmic reticulum stress rather than ATP depletion. Cancer Chemother Pharm..

[30] Tanida I, Ueno T, Kominami E (2008). LC3 and Autophagy. Methods Mol Biol..

[31] Kang R, Zeh HJ, Lotze MT, Tang D (2011). The Beclin 1 network regulates autophagy and apoptosis. Cell Death Differ..

[32] Sun Y, Xia M, Yan H, Han Y, Zhang F, Hu Z (2018). Berberine attenuates hepatic steatosis and enhances energy expenditure in mice by inducing autophagy and fibroblast growth factor 21. Br J Pharm..

[33] Robinson GL, Dinsdale D, Macfarlane M, Cain K (2012). Switching from aerobic glycolysis to oxidative phosphorylation modulates the sensitivity of mantle cell lymphoma cells to TRAIL. Oncogene.

[34] Kim J, Kundu M, Viollet B, Guan KL (2011). AMPK and mTOR regulate autophagy through direct phosphorylation of Ulk1. Nat Cell Biol..

[35] González A, Hall MN, Lin SC, Hardie DG, AMPK (2020). and TOR: the Yin and Yang of Cellular Nutrient Sensing and Growth Control. Cell Metab..

[36] Zhang D, Li P, Gao Y, Song Y, Zhu Y, Su H (2021). Discovery of a Candidate Containing an (S)-3,3-Difluoro-1-(4-methylpiperazin-1-yl)-2,3-dihydro-1H-inden Scaffold as a Highly Potent Pan-Inhibitor of the BCR-ABL Kinase Including the T315I-Resistant Mutant for the Treatment of Chronic Myeloid Leukemia. J Med Chem..

[37] Singh D, Banerji AK, Dwarakanath BS, Tripathi RP, Gupta JP, Mathew TL (2005). Optimizing cancer radiotherapy with 2-deoxy-d-glucose dose escalation studies in patients with glioblastoma multiforme. Strahlenther Onkol..

[38] Chou TC (2006). Theoretical basis, experimental design, and computerized simulation of synergism and antagonism in drug combination studies. Pharm Rev..

