# Supplementary figures and images for: Modulation of energy metabolism to overcome drug resistance in chronic myeloid leukemia cells through induction of autophagy
Source: Cell Death Discov. 2022 Apr 20;8:212. doi: 10.1038/s41420-022-00991-w (PMC9021256; doi:10.1038/s41420-022-00991-w)

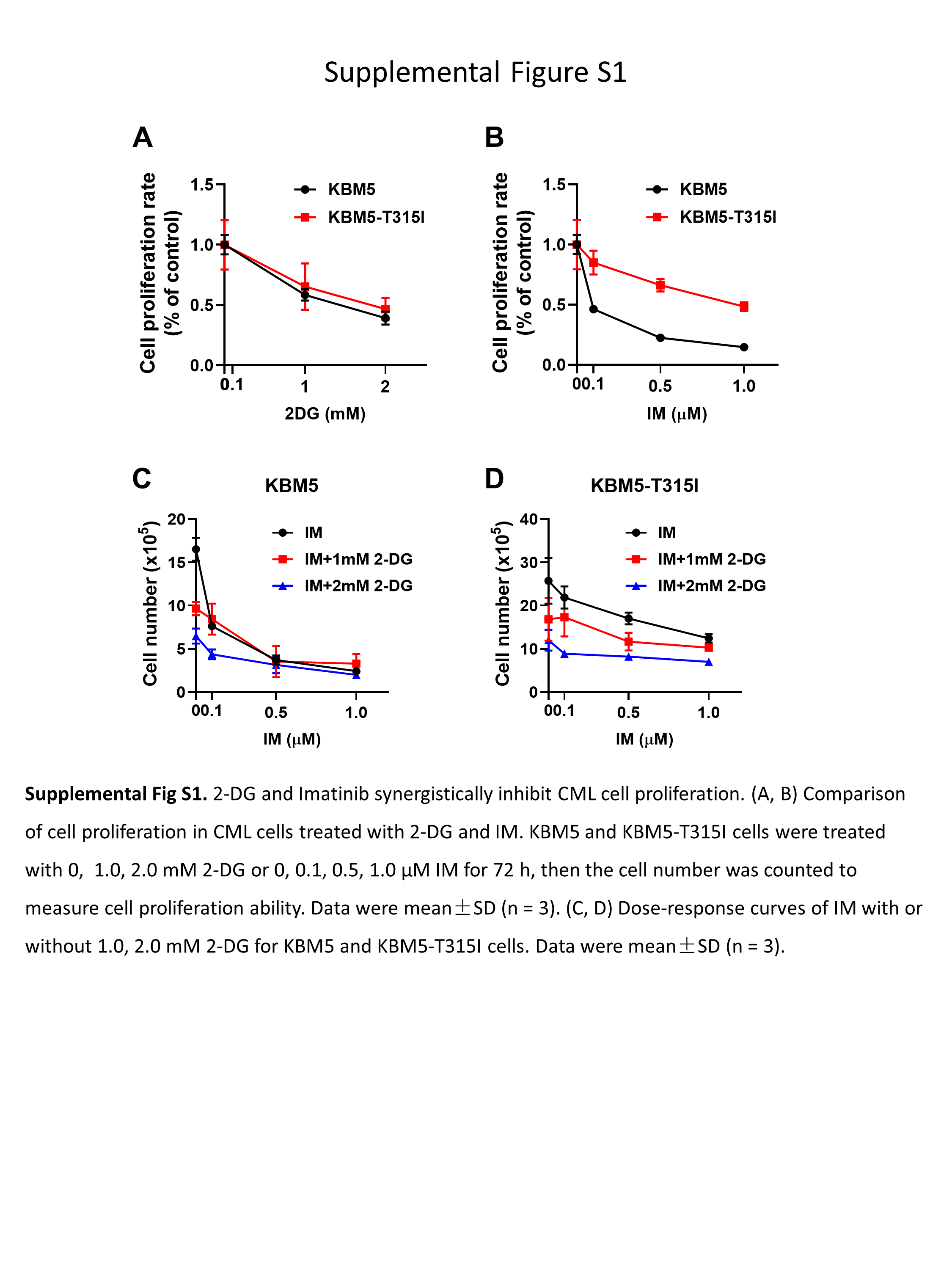

Supplement: Supplementary file 1 — Supplemental Figure S1 [file 41420_2022_991_MOESM1_ESM.tif]

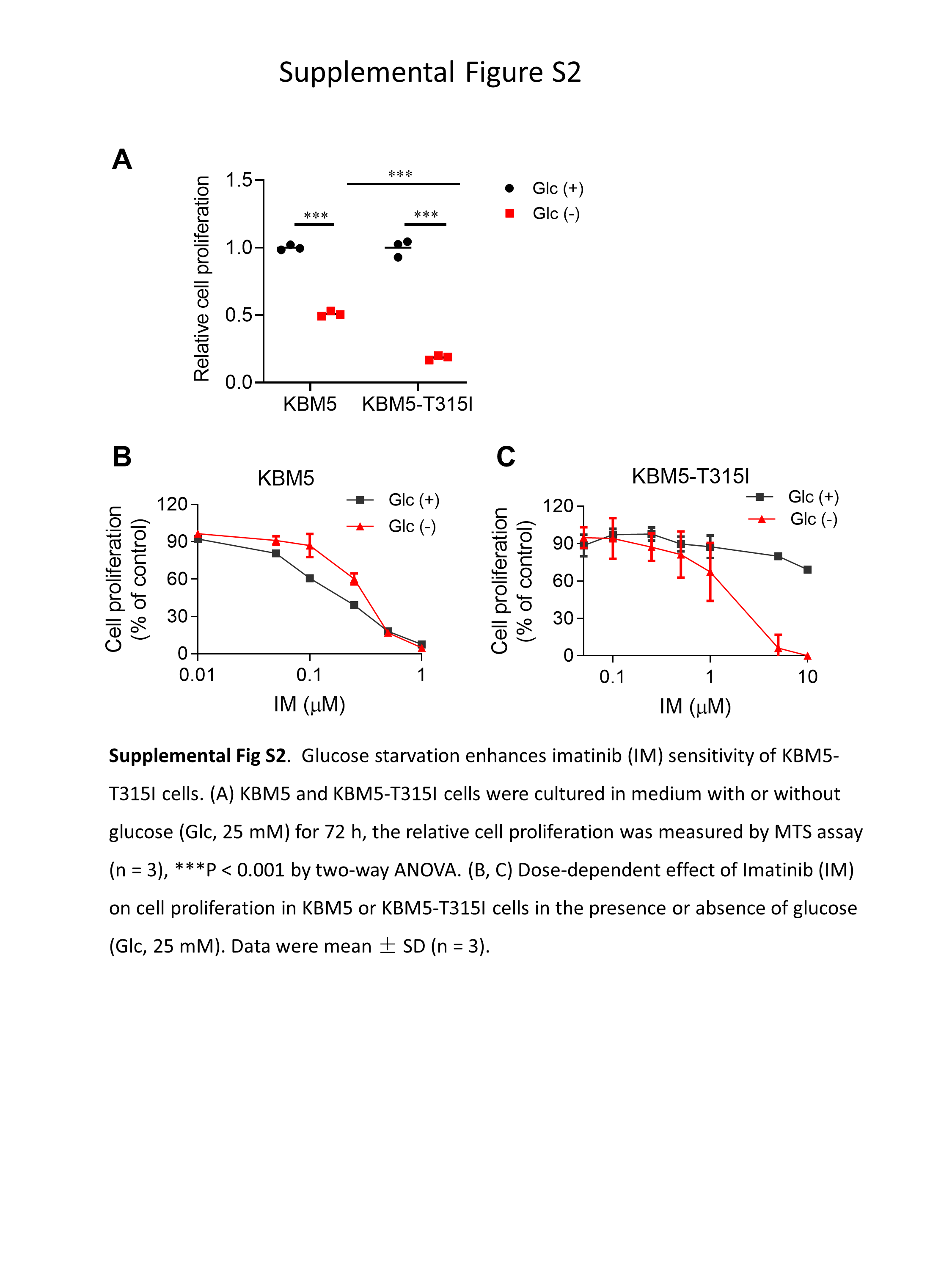

Supplement: Supplementary file 2 — Supplemental Figure S2 [file 41420_2022_991_MOESM2_ESM.tif]

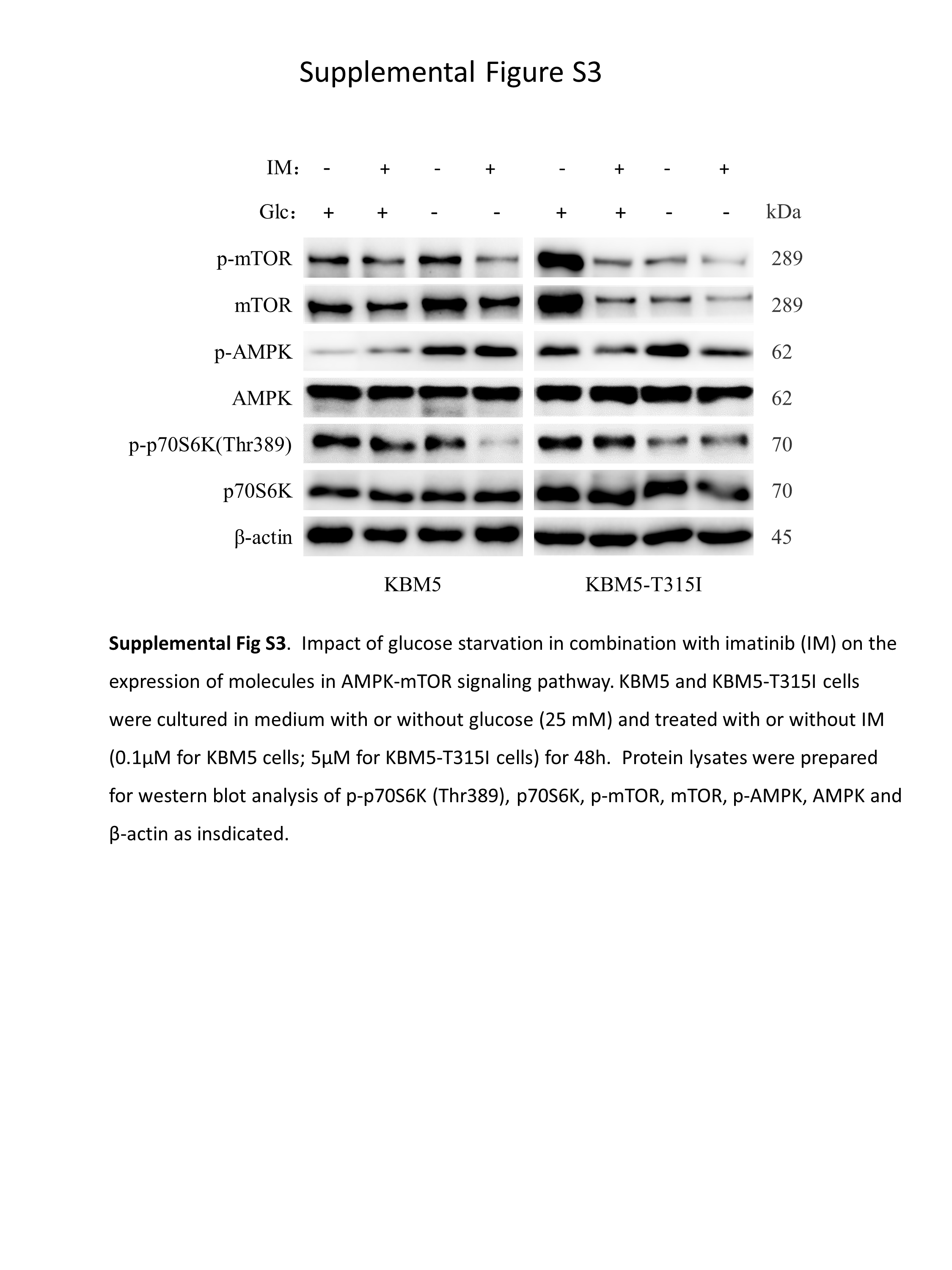

Supplement: Supplementary file 3 — Supplemental Figure S3 [file 41420_2022_991_MOESM3_ESM.tif]

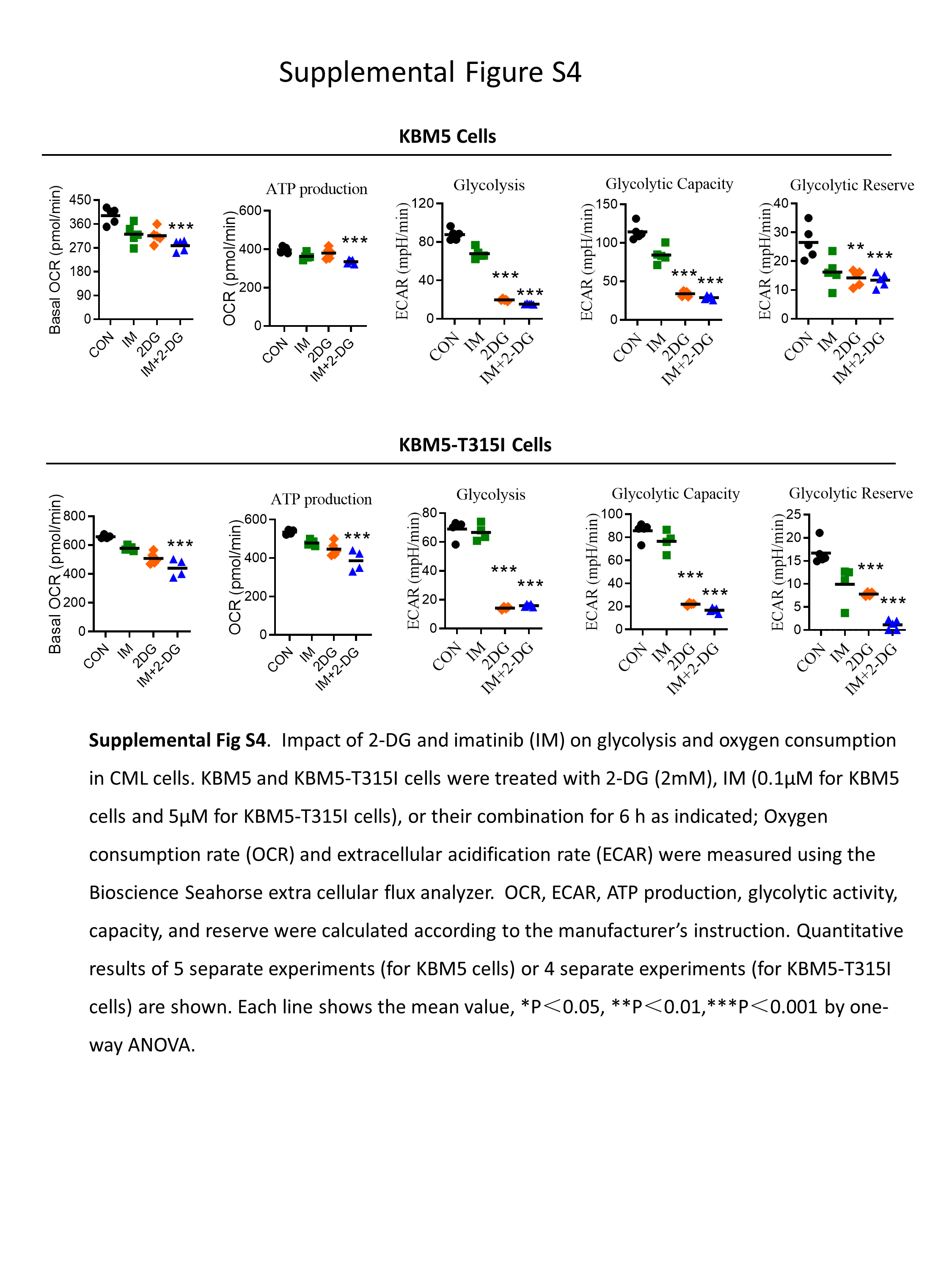

Supplement: Supplementary file 4 — Supplemental Figure S4 [file 41420_2022_991_MOESM4_ESM.tif]
