# Supplementary material for: Modulation of energy metabolism to overcome drug resistance in chronic myeloid leukemia cells through induction of autophagy
Source: Cell Death Discov. 2022 Apr 20;8:212. doi: 10.1038/s41420-022-00991-w (PMC9021256; doi:10.1038/s41420-022-00991-w)

Figure 2B

2-DG(mM)

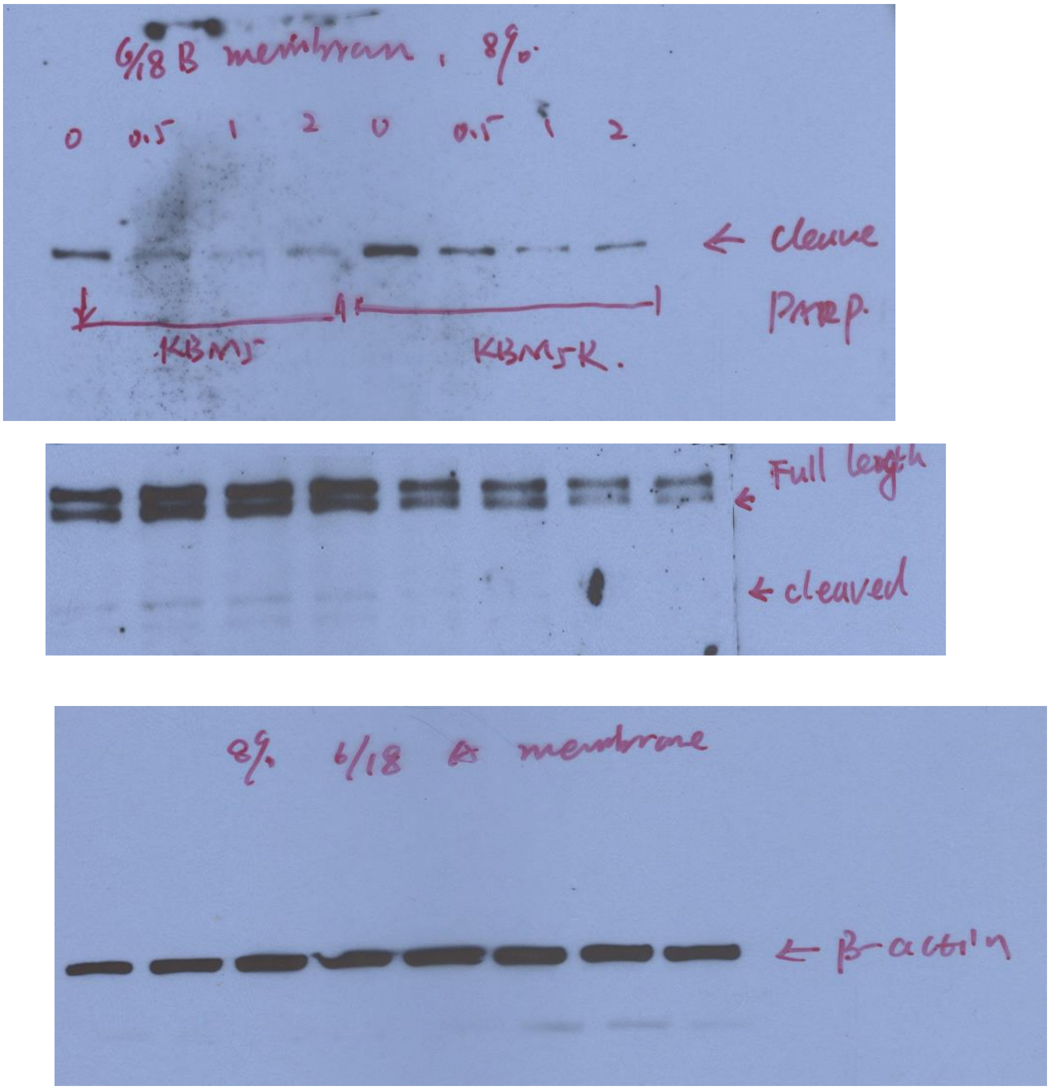

Note：KBM5R=KBM5-T315I (Imatinib-resistant mutant)

Figure 3A

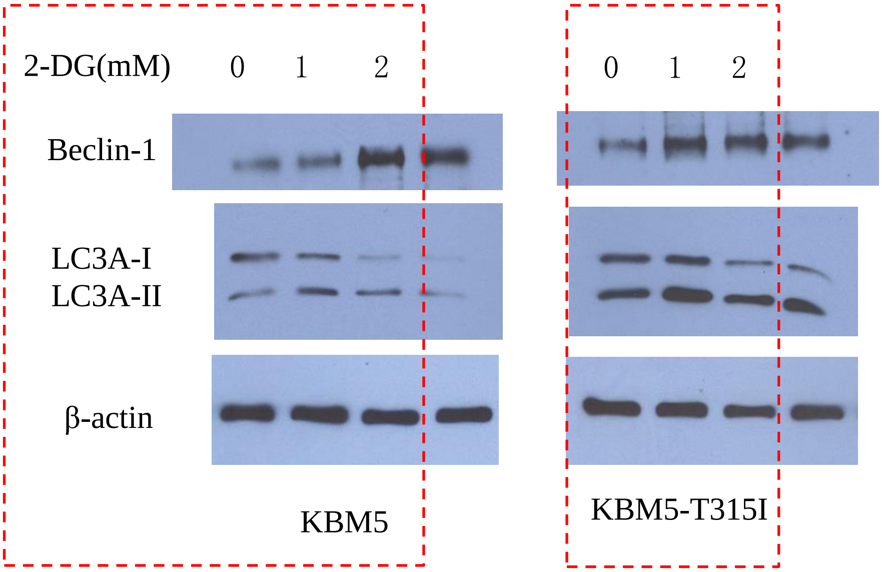

Figure 3B

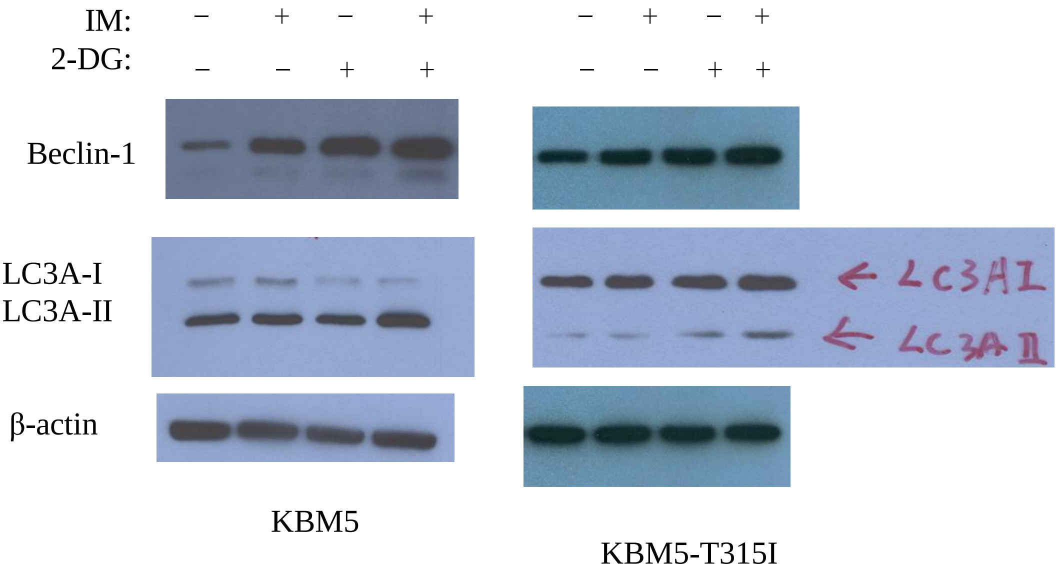
IM: – + – + – + – +
2-DG: – – + + – – + +
Beclin-1
LC3A-I
LC3A-II
β-actin
LC3A I
LC3A II
KBM5
KBM5-T315I

Figure 3C

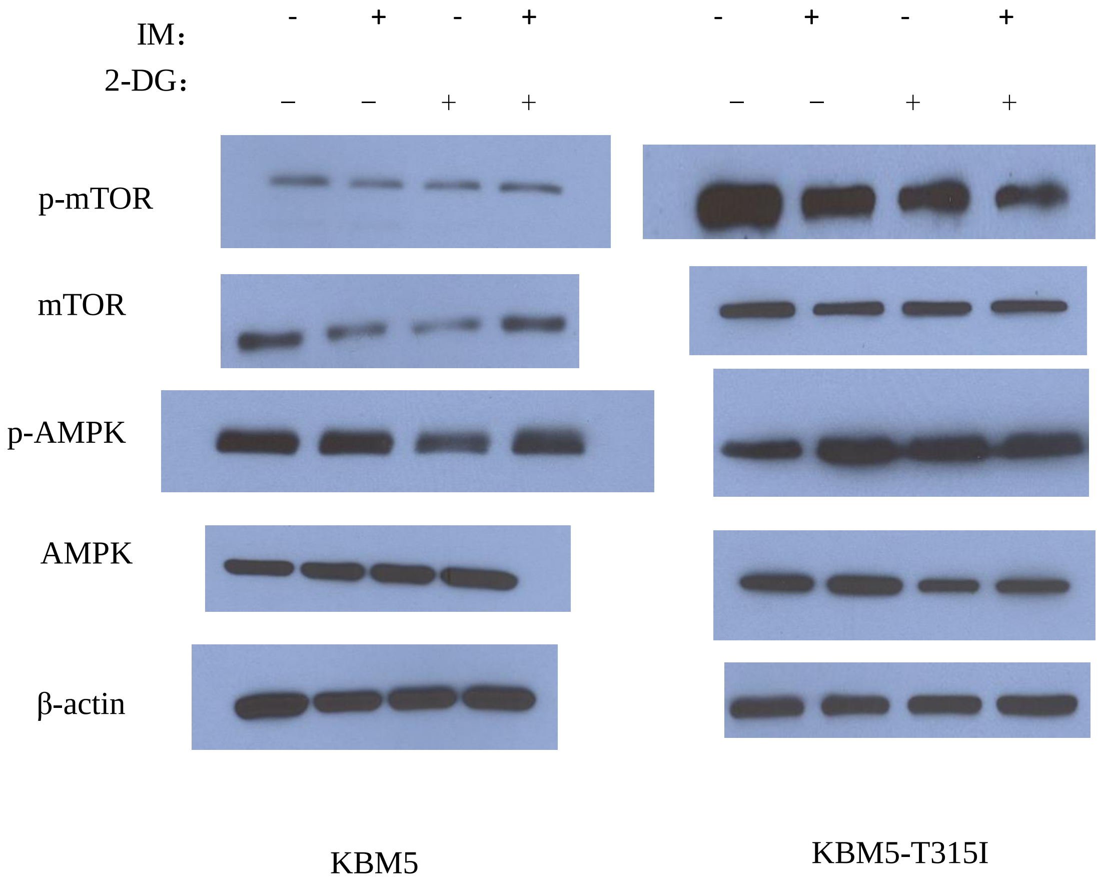

Figure 3D

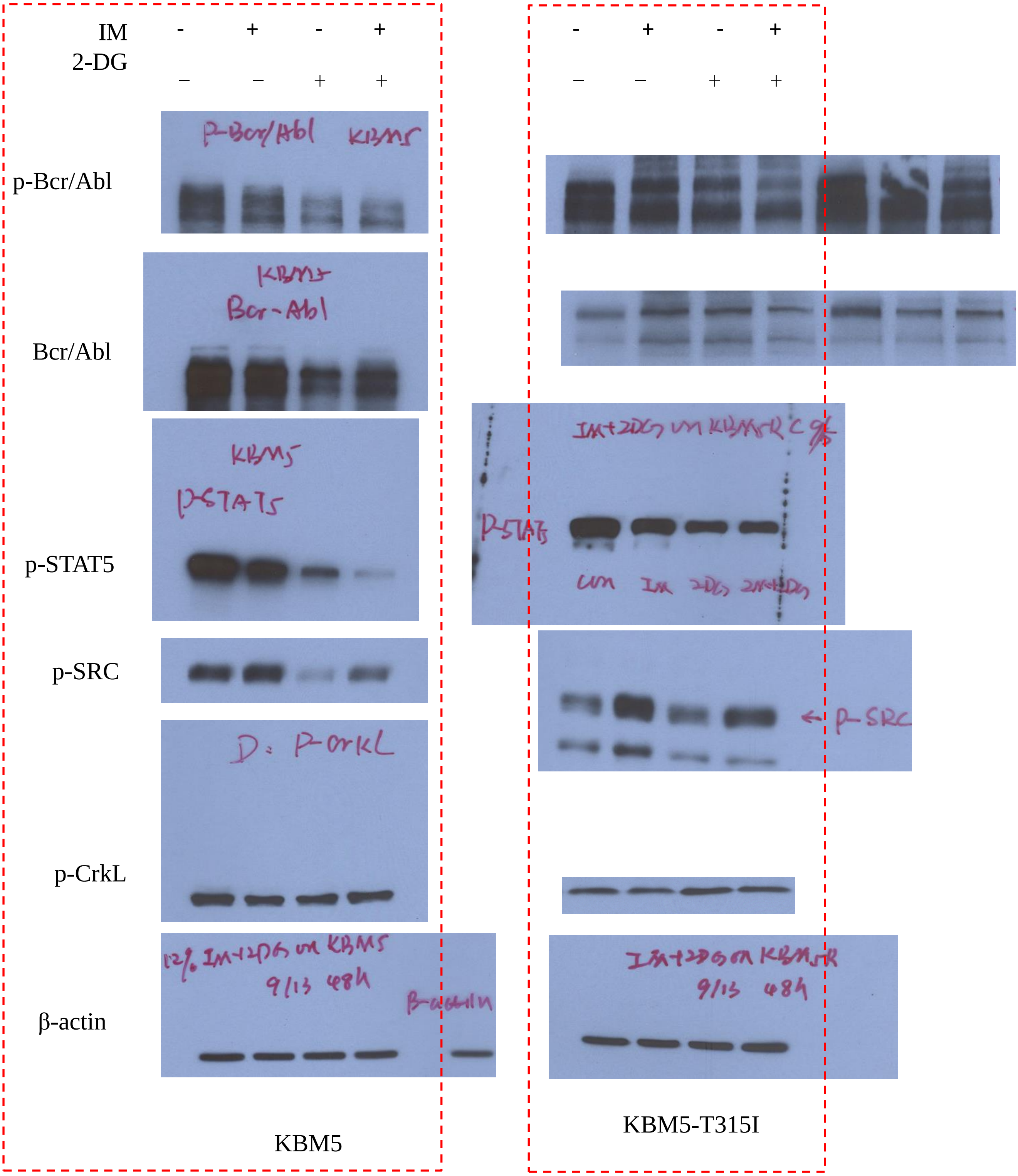
IM - + - +
2-DG − − + +
p-Bcr/Abl
Bcr/Abl
p-STAT5
p-SRC
p-CrkL
β-actin
KBM5
- + - +
− − + +
KBM5-T315I

Figure 5A

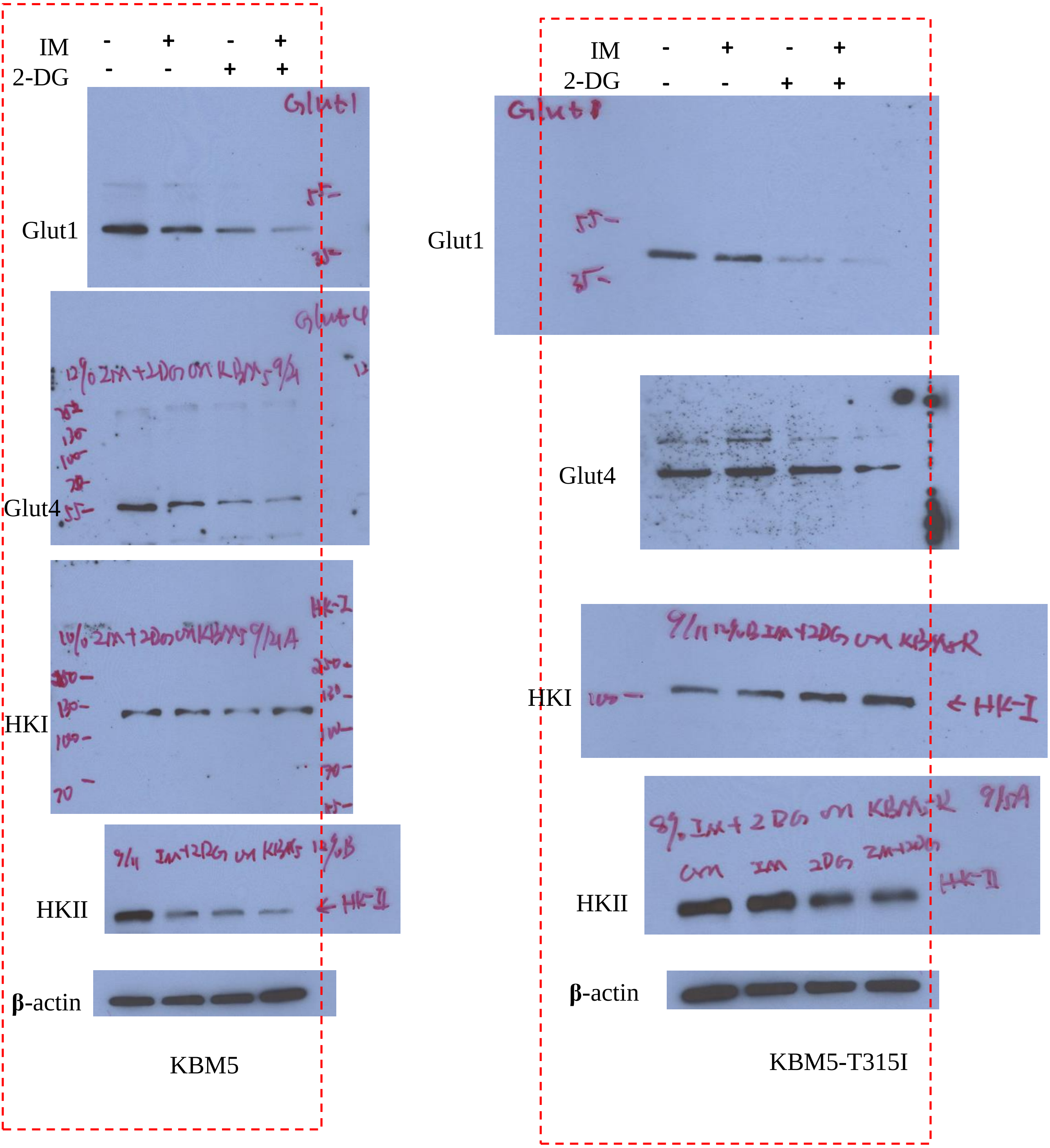
IM - + - +
2-DG - - + +
Glut1
Glut4
HKI
HKII
β-actin
KBM5
IM - + - +
2-DG - - + +
Glut1
Glut4
HKI
HKII
β-actin
KBM5-T315I

Figure 5B

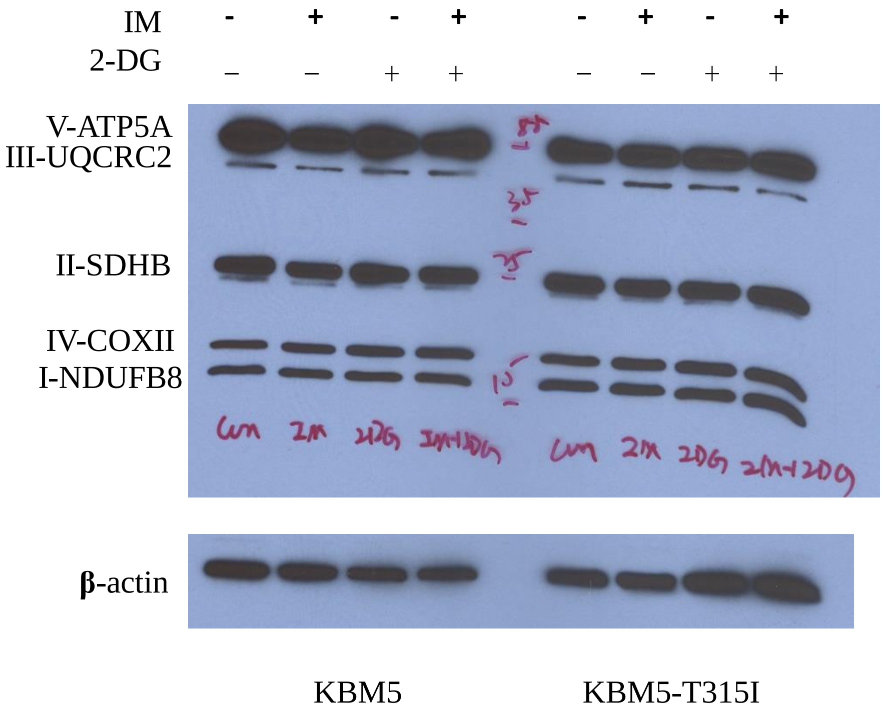
IM - + - + - + - +
2-DG – – + + – – + +
V-ATP5A
III-UQCRC2
II-SDHB
IV-COXII
I-NDUFB8
β-actin
KBM5
KBM5-T315I

Supplemental Figure S3

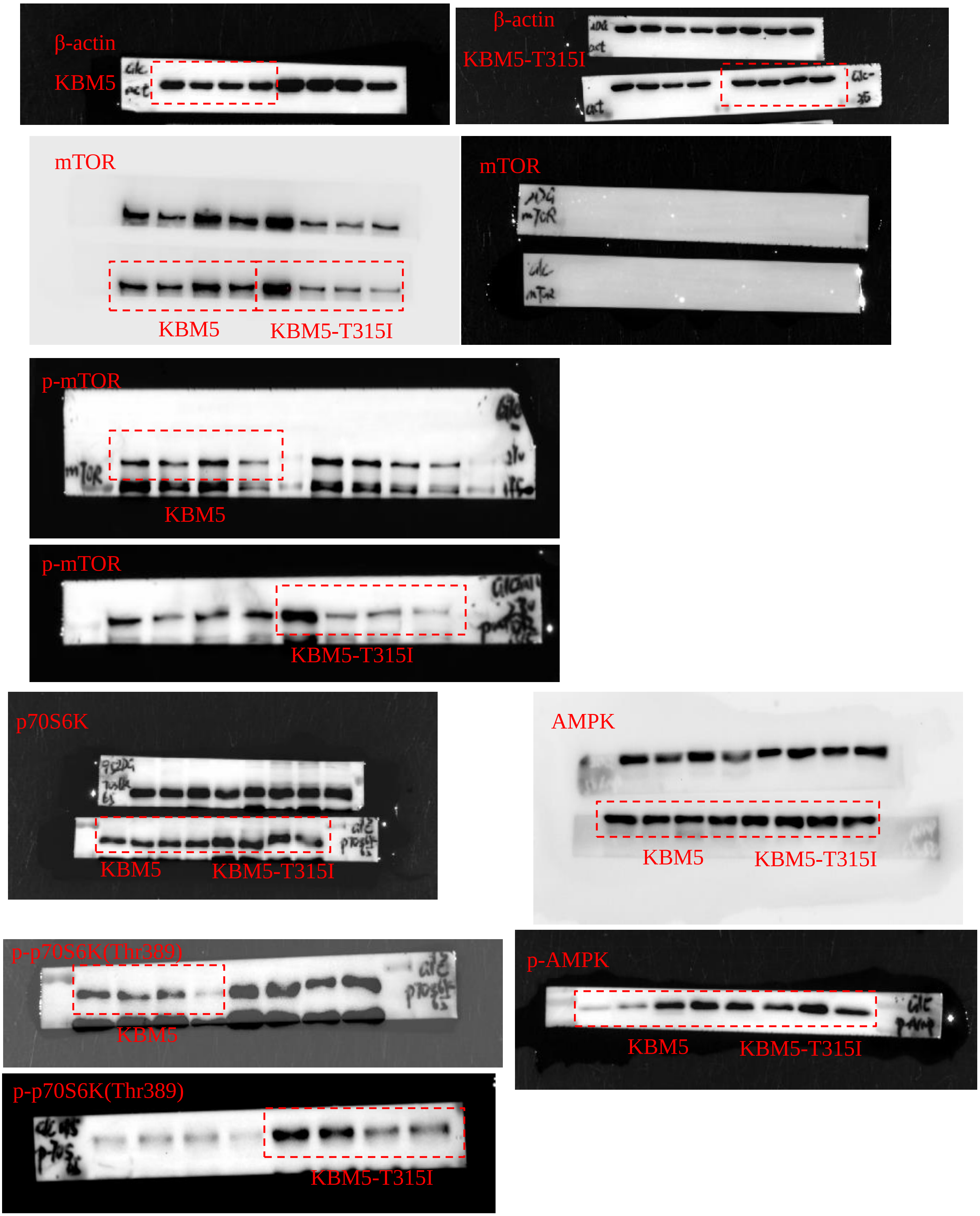

Supplement: Supplementary file 5 — Supplemental material-original western blots [file 41420_2022_991_MOESM5_ESM.pdf]
